# *DPP4* gene variation affects GLP-1 secretion, insulin secretion, and glucose tolerance in humans with high body adiposity

**DOI:** 10.1371/journal.pone.0181880

**Published:** 2017-07-27

**Authors:** Anja Böhm, Robert Wagner, Fausto Machicao, Jens Juul Holst, Baptist Gallwitz, Norbert Stefan, Andreas Fritsche, Hans-Ulrich Häring, Harald Staiger

**Affiliations:** 1 Department of Internal Medicine, Division of Endocrinology, Diabetology, Angiology, Nephrology and Clinical Chemistry, Eberhard Karls University Tübingen, Tübingen, Germany; 2 German Center for Diabetes Research (DZD), Tübingen, Germany; 3 Institute for Diabetes Research and Metabolic Diseases of the Helmholtz Centre Munich at the University of Tübingen, Tübingen, Germany; 4 Section of Translational Metabolic Physiology, Novo Nordisk Foundation Center for Basic Metabolic Research, University of Copenhagen, Copenhagen, Denmark; 5 Department of Internal Medicine, Division of Nutritional and Preventive Medicine, Eberhard Karls University Tübingen, Tübingen, Germany; 6 Interfaculty Center for Pharmacogenomics and Pharma Research at the Eberhard Karls University Tübingen, Tübingen, Germany; 7 Institute of Pharmaceutical Sciences, Department of Pharmacy and Biochemistry, Eberhard Karls University Tübingen, Tübingen, Germany; Baylor College of Medicine, UNITED STATES

## Abstract

**Objective:**

Dipeptidyl-peptidase 4 (DPP-4) cleaves and inactivates the insulinotropic hormones glucagon-like peptide 1 (GLP-1) and gastric inhibitory polypeptide, collectively termed incretins. DPP-4 inhibitors entered clinical practice as approved therapeutics for type-2 diabetes in 2006. However, inter-individual variance in the responsiveness to DPP-4 inhibitors was reported. Thus, we asked whether genetic variation in the *DPP4* gene affects incretin levels, insulin secretion, and glucose tolerance in participants of the TÜbingen Family study for type-2 diabetes (TÜF).

**Research design and methods:**

Fourteen common (minor allele frequencies ≥0.05) *DPP4* tagging single nucleotide polymorphisms (SNPs) were genotyped in 1,976 non-diabetic TÜF participants characterized by oral glucose tolerance tests and bioimpedance measurements. In a subgroup of 168 subjects, plasma incretin levels were determined.

**Results:**

We identified a variant, i.e., SNP rs6741949, in intron 2 of the *DPP4* gene that, after correction for multiple comparisons and appropriate adjustment, revealed a significant genotype-body fat interaction effect on glucose-stimulated plasma GLP-1 levels (p = 0.0021). Notably, no genotype-BMI interaction effects were detected (p = 0.8). After stratification for body fat content, the SNP negatively affected glucose-stimulated GLP-1 levels (p = 0.0229), insulin secretion (p = 0.0061), and glucose tolerance (p = 0.0208) in subjects with high body fat content only.

**Conclusions:**

A common variant, i.e., SNP rs6741949, in the *DPP4* gene interacts with body adiposity and negatively affects glucose-stimulated GLP-1 levels, insulin secretion, and glucose tolerance. Whether this SNP underlies the reported inter-individual variance in responsiveness to DPP-4 inhibitors, at least in subjects with high body fat content, remains to be shown.

## Introduction

Dipeptidyl-peptidase 4 (DPP-4, alias CD26) is a ubiquitously expressed single-pass type II transmembrane protein that aggregates in cholesterol-rich lipid rafts and interacts with several other proteins, e.g., caveolin 1, adenosine deaminase, fibroblast activation protein, insulin-like growth factor 2 receptor, receptor-type protein tyrosine phosphatase C, and extracellular matrix proteins [1]. The homodimer represents a proteolytically active enzyme (EC 3.4.14.5) that cleaves N-terminal X-proline and X-alanine dipeptides from polypeptides with unsubstituted N-termini [2]. Among its known substrates are chemokines, growth factors, neuropeptides, and peptide hormones, such as the incretins glucagon-like peptide 1 (GLP-1) and gastric inhibitory polypeptide (GIP) [1,3]. Cleavage of the incretins by DPP-4 results in loss of these hormones’ insulinotropic activities and initiates their degradation [4]. A soluble form of DPP-4 is known to be present in human plasma, urine, and seminal fluid and is thought to derive from proteolytic cleavage of the transmembrane protein [5]. The regulation of this process is however poorly understood.

DPP-4 exerts pleiotropic functions, e.g., in metabolism, immune reactions, and cancer growth [6]. With respect to glucose metabolism, DPP-4 deficiency in rodent models was shown to improve glucose tolerance and insulin sensitivity via enhanced glucose-stimulated insulin secretion, probably due to increased circulating GLP-1 levels, and to confer resistance to high-fat diet-induced body weight gain and hyperinsulinaemia as a result of reduced food intake and increased energy expenditure [7–9]. Since pancreatic β-cell failure is a hallmark of type-2 diabetes [10,11], a very promising strategy, intensely followed by pharmaceutical companies, to fight the disease is to improve β-cell function with the help of drugs that enhance the incretin axis. With regard to the negative impact of DPP-4 on incretin levels and activities, two options were conceivable: (i) application of DPP-4-resistant GLP-1 analogues or mimetics with prolonged half-lives; and (ii) augmentation of endogenous incretin levels by DPP-4 inhibition [12]. With the GLP-1 analogues [13], such as exenatide and liraglutide, and the DPP-4 inhibitors collectively termed ‘gliptins’ [14], such as sitagliptin, vildagliptin, linagliptin, and saxagliptin, both approaches have found the way into clinical practice. While these drugs represent valuable anti-diabetic therapeutic options from a statistical point of view, very recent studies report considerable biological variance between individuals in the responsiveness to DPP-4 inhibitors [15–17]. The reasons for good versus diminished response to these drugs are however largely unknown.

In this study, we therefore asked whether common genetic variation [minor allele frequency (MAF) ≥0.05] in the *DPP4* gene exists that affects incretin levels, insulin secretion, and glucose tolerance in non-diabetic individuals recruited from the TÜbingen Family study for type 2 diabetes (TÜF). The identification of single nucleotide polymorphisms (SNPs) which determine differences in the aforementioned parameters could, after further corroboration in pharmacogenetic settings, support clinical decisions in terms of individualized therapy: for instance, subjects who do not adequately respond to DPP-4 inhibitors due to genetically increased DPP-4 levels/activities would possibly better benefit from DPP-4-resistant incretin mimetics.

## Materials and methods

### Ethics statement

All participants gave informed written consent to the study which adhered to the Declaration of Helsinki. The study protocol was approved by the Ethics Committee of the Eberhard Karls University Tübingen.

### Subjects

The overall study population consisted of 1,976 White Europeans from the Southwest of Germany and was recruited from the ongoing TÜF study. TÜF currently comprises more than 3,000 non-related individuals at increased risk for type-2 diabetes, i.e., non-diabetic subjects with a family history of type 2 diabetes, a BMI ≥27 kg/m2, impaired fasting glycaemia, and/or previous gestational diabetes [18]. All TÜF participants underwent assessment of medical history, smoking status, and alcohol consumption habits; the subjects furthermore agreed to undergo physical examination, routine blood tests, and oral glucose tolerance tests (OGTTs). In the overall study population, only individuals with complete phenotypic data sets and documented absence of medication known to influence glucose tolerance, insulin sensitivity, or insulin secretion were included. In a randomly selected subgroup of 168 subjects having blood stored in the presence of protease inhibitor, plasma levels of the incretins GLP-1 and GIP were determined. The clinical characteristics of the overall study population and the GLP-1/GIP subgroup are presented in Table 1.

**Table 1:** Clinical characteristics of the study population.

	Overall study population		GLP-1/GIP subgroup	
Sample size (N)	1,976		168	
Women / men (N)	1,306 / 670		114 / 54	
NGT / IFG / IGT / IFG&IGT (N)	1,392 / 223 / 194 / 167		113 / 24 / 13 / 18	
	Median	IQR	Median	IQR
Age (y)	39	29–50	47	37–54
BMI (kg/m^2^)	27.6	23.5–34.5	29.4	26.7–34.9
Body fat (%)	31	23–41	33	27–39
Glucose_0_ OGTT (mmol/L)	5.11	4.78–5.44	5.28	4.89–5.59
Glucose_120_ OGTT (mmol/L)	6.20	5.17–7.28	6.44	5.56–7.49
ISI OGTT (*10^15^ L^2^*mol^-2^)	12.6	7.4–20.7	11.8	7.3–16.0
AUC Ins_0-30_/AUC Glc_0-30_ OGTT (*10^−9^)	36.1	23.3–56.5	38.3	22.8–57.5
AUC Cpep_0-120_/AUC Glc_0-120_ OGTT (*10^−9^)	308	248–376	291	231–361
GLP-1_0_ OGTT (pmol/L)	-	-	17	12–22
GIP_0_ OGTT (pmol/L)	-	-	15	9–19

Medians and IQRs of the continous traits are given for the overall study population and the GLP-1/GIP subgroup unstratified for gender or glucose tolerance status. AUC–area under the curve; BMI–body mass index; Cpep–C-peptide; GIP–gastric inhibitory polypeptide; Glc–glucose; GLP-1 –glucagon-like peptide 1; IFG–impaired fasting glycaemia; Ins–insulin; IGT–impaired glucose tolerance; IQR–interquartile range; ISI–insulin sensitivity index; NGT–normal glucose tolerance; OGTT–oral glucose tolerance test

### OGTT

A standardized 75-g OGTT was performed following a 10-h overnight fast. For the determination of plasma glucose, insulin, and C-peptide levels, venous blood samples were drawn at baseline and at time-points 30, 60, 90, and 120 min of the OGTT [18]. Incretin levels were measured at baseline and at time-points 30 and 120 min.

### Measurements of body fat content

BMI as a crude proxy for body fat content was calculated as weight divided by squared height (in kg/m^2^). The percentage of body fat was measured by bioelectrical impedance (BIA-101, RJL systems, Detroit, MI, USA).

### Laboratory measurements

Plasma glucose levels (in mmol/L) were measured with a bedside glucose analyzer (glucose oxidase method, Yellow Springs Instruments, Yellow Springs, OH, USA). Plasma insulin and C-peptide levels (in pmol/L, both) were determined by commercial chemiluminescence assays for ADVIA Centaur (Siemens Medical Solutions, Fernwald, Germany). Total GLP-1 and GIP levels (in pmol/L, both) were quantified using radioimmunoassays specific for the C-terminal parts of the peptides [19,20]. To avoid incretin degradation, venous blood was drawn into chilled tubes containing EDTA and aprotinin (400 kallikrein inhibitor units/mL blood; Bayer, Leverkusen, Germany) and kept on ice. After centrifugation at 4°C, plasma for hormone analyses was kept frozen at -20°C.

### Calculations

The OGTT-derived insulin sensitivity index (ISI OGTT) was estimated as proposed earlier [21]: 10,000/[c(Glc_0_)*c(Ins_0_)*c(Glc_mean_)*c(Ins_mean_)]½ (with c = concentration, Glc = glucose, and Ins = insulin). OGTT-derived insulin secretion was estimated as area under the curve (AUC) Cpep_0-120_/AUC Glc_0-120_ according to the trapezoid method: ½[½c(Cpep_0_)+c(Cpep_30_)+c(Cpep_60_)+c(Cpep_90_)+½c(Cpep_120_)]/½[½c(Glc_0_)+c(Glc_30_)+c(Glc_60_)+c(Glc_90_)+½c(Glc_120_)] (with Cpep = C-peptide). This insulin secretion index was recently shown to be superior to several fasting state- and OGTT-derived indices for the detection of genetically determined defects in the incretin axis [22].

### Selection of tagging SNPs

**Fig 1:**
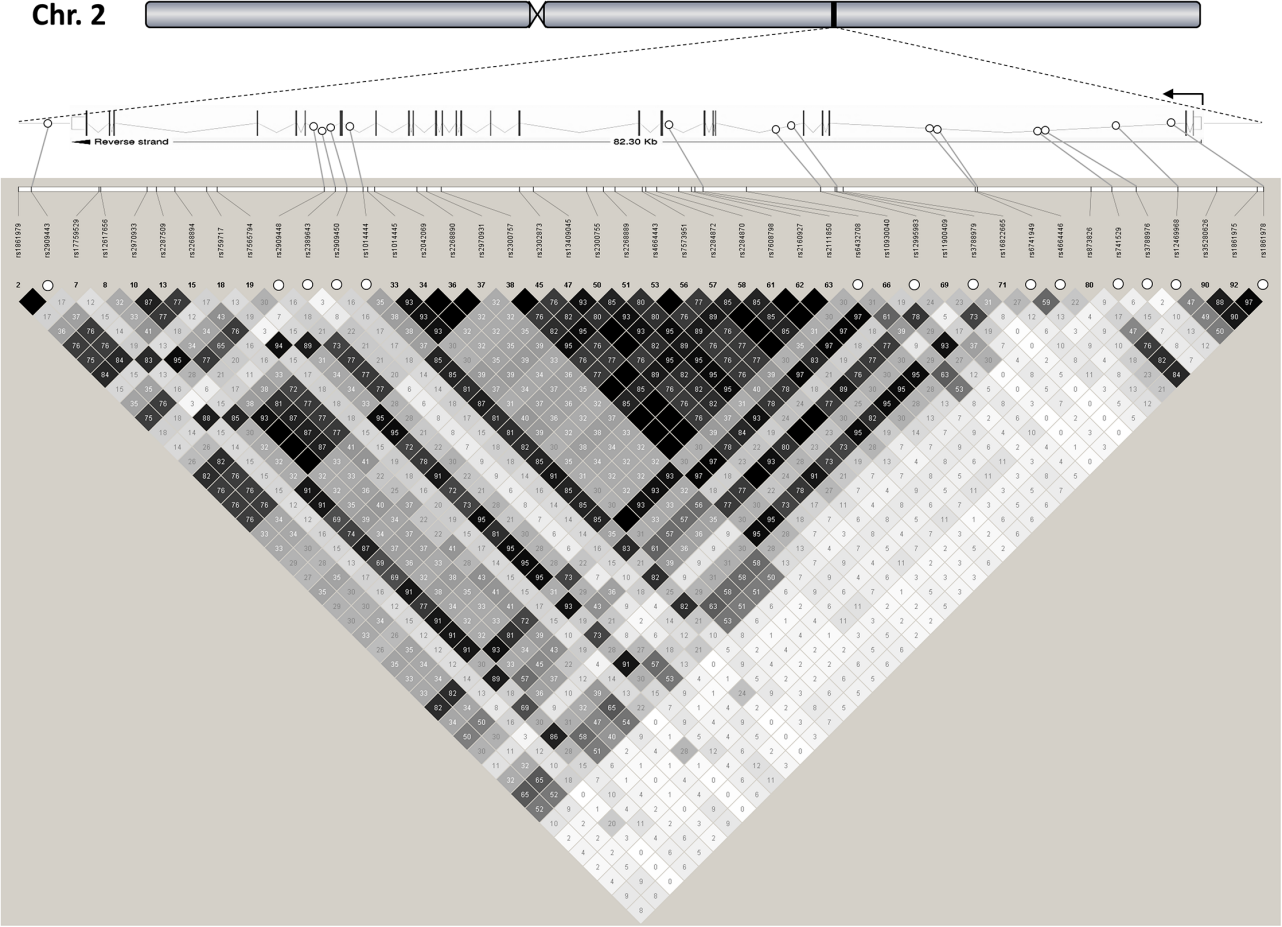
*DPP4* gene locus on human chromosome 2q24.2 and tagging SNPs. The *DPP4* gene is located on the reverse strand, consists of 26 exons and 25 introns, and spans 82.3 kb from nucleotide position 162,848,755 to nucleotide position 162,931,052 (Ensembl reference data). A region from 5 kb upstream to 5 kb downstream of the gene was analyzed. This genomic region does not overlap with other known gene loci. The 44 common HapMap SNPs of the locus are shown (minor allele frequencies ≥0.05). The locations of the 14 tagging SNPs are highlighted by white circles. HapMap-derived linkage disequilibrium data (r^2^x100) are indicated in shaded diamonds (black diamond without value stands for r^2^ = 1.0). SNP–single nucleotide polymorphism.

Based on publicly available data of the International HapMap Project (phase III) derived from the Central European (CEU) population (release #28 August 2010, http://hapmap.ncbi.nlm.nih.gov/index.html.en), we analyzed *in silico* a genomic area on human chromosome 2q24.2 spanning the *DPP4* gene (82.3 kb, 26 exons, 25 introns, located on the reverse strand), 5 kb of the gene’s 5’-flanking region, and 5 kb of its 3’-flanking region (Fig 1). The *DPP4* locus is flanked ~70 kb upstream by the *GCG* gene (encoding proglucagon, the source of glucagon, GLP-1, and GLP-2) and ~7 kb downstream by the *SLC4A10* gene (encoding a sodium-dependent chloride/bicarbonate exchanger with no known function in glucose metabolism). Notably, no obvious high-linkage disequilibrium (LD) block overlapping the screened *DPP4* locus and one of its adjacent genes was detected. Within the analyzed *DPP4* locus, 44 informative HapMap SNPs with Hardy-Weinberg p-values and MAFs ≥0.05 were found, and all of them are intronic or located in the 3’-flanking region. Their LD data (r^2^-values from HapMap) are presented in Fig 1. Using the tagger analysis tool of the Haploview freeware (http://www.broadinstitute.org/scientific-community/science/programs/medical-and-population-genetics/haploview/haploview), 14 tagging SNPs were identified covering all the other common HapMap SNPs with an r^2^ ≥0.8 (100% coverage of the common genetic variation in the locus). These tagging SNPs were rs2909443 (A/G) in the 3’-flanking region, rs2909448 (T/C), rs2389643 (C/T), and rs2909450 (G/A) in intron 20, rs1014444 (A/G) in intron 19, rs6432708 (T/C) in intron 8, rs12995983 (T/C) and rs3788979 (G/A) in intron 5, and rs6741949 (G/C), rs4664446 (A/G), rs741529 (G/A), rs3788976 (C/T), rs12469968 (A/G), and rs1861978 (T/G) in intron 2 (Fig 1).

### Genotyping

DNA was isolated from whole blood using a commercial kit (NucleoSpin, Macherey & Nagel, Düren, Germany). The 14 *DPP4* tagging SNPs were genotyped using the mass spectrometry system massARRAY from Sequenom and the manufacturer’s iPLEX software (Sequenom, Hamburg, Germany). The call rates were ≥98.9%. The mass spectrometric results were validated in 50 randomly selected subjects by bidirectional sequencing, and both methods gave 100% identical results.

### Statistical analyses

Hardy-Weinberg equilibrium was tested using χ^2^ test (based on one degree of freedom). LD (D’-, r^2^-values) between the tagging SNPs was analyzed using the MIDAS 1.0 freeware (http://www.genes.org.uk/software/midas). Continuous variables with non-normal distribution were log_*e*_-transformed prior to statistical analysis. Multiple linear regression analysis was performed using the least-squares method. In the regression models, the trait of interest (incretin level, insulin secretion, or glucose level) was chosen as outcome variable, the SNP genotype (in the additive inheritance model) as independent variable, and gender, age, and percentage of body fat (and ISI OGTT when testing SNP associations with insulin secretion) as confounding variables. In some analyses, glucose tolerance status was included in the multiple regression models as additional confounding variable in form of a dummy variable (normal glucose tolerance = 0, impaired fasting glycaemia = 1, impaired glucose tolerance = 2, and impaired fasting glycaemia + impaired glucose tolerance = 3). For analysis of SNP-body fat interaction effects on incretin levels, the respective cross effects were tested with gender and age as confounding variables. When testing all 14 tagging SNPs in parallel, a Bonferroni-corrected p-value <0.0037 was considered statistically significant. In all subsequent follow-up analyses testing only SNP rs6741949, the significance threshold was set at p<0.05. We did not correct for the tested metabolic traits since these were not independent. For all analyses, the statistical software JMP 8.0 (SAS Institute, Cary, NC, USA) was used. Based on the observed MAFs, the study was sufficiently powered (1-β≥0.8) to detect SNP effects on insulin secretion ≥5% in the overall population and to detect SNP effects on fasting GLP-1 levels ≥26% in the GLP-1/GIP subgroup (two-sided p<0.05). Power calculations were performed using the Quanto 1.2.4 freeware (http://hydra.usc.edu/gxe).

### Data availability

Data are available upon request from the principal investigators of the TÜF study. Please contact us at http://www.med.uni-tuebingen.de/Mitarbeiter/Kliniken/Medizinische+Klinik/Innere+Medizin+IV/Forschung+/Klinisches+Studienzentrum.html. The volume and complexity of the data collected precludes public data deposition because the participants could be identifiable from such extensive data which would compromise participants’ privacy.

## Results

On average, the overall study population was young (median age 39 y) and overweight (median BMI 27.6 kg/m^2^), two thirds were women and one third men (Table 1). Seventy percent of the subjects had normal glucose tolerance, whereas the remaining 30% were prediabetic having impaired fasting glycaemia and/or impaired glucose tolerance (Table 1). In the GLP-1/GIP subgroup, the medians of age and BMI were slightly higher (47 y and 29.4 kg/m^2^, respectively), the gender distribution and the proportion of prediabetic individuals were however similar to the overall study population (Table 1). Furthermore, no major differences were observed between the overall study population and the GLP-1/GIP subgroup with respect to measures of insulin sensitivity and insulin secretion (Table 1).

All 14 tagging SNPs, covering 100% of the common (MAF ≥0.05) genetic variants in the *DPP4* locus, were genotyped in the overall study population (for locations and HapMap LD data, see Fig 1). All but one of the tagging SNPs obeyed Hardy-Weinberg equilibrium (p≥0.05). SNP rs4664446 was not in Hardy-Weinberg equilibrium (p = 0.03). Since no genotyping error could be found, we included SNP rs4664446 in our correlational analyses. The MAFs observed in our study population were similar to those reported for the HapMap CEU population (S1 Table). Interestingly, the two SNPs with MAFs closest to 0.5, i.e., rs4664446 and rs12469968, revealed inverted allele distributions: for both SNPs, the minor allele was the G-allele in our study, whereas it was the A-allele in the HapMap CEU population. Based on the observed LD data (r^2^-values), the linkage between the tagging SNPs ranged from very low (r^2^<0.001) to moderate (r^2^ = 0.613) with the following exception: the three SNPs rs2909443, rs2909448, and rs64322708 were in high linkage in our population (r^2^-values among all three >0.8; S2 Table). By contrast, the r^2^-values between SNPs rs2909443 and rs2909448 as well as between SNPs rs2909448 and rs6432708 did not reach 0.8 in the HapMap CEU population (Fig 1).

**Fig 2:**
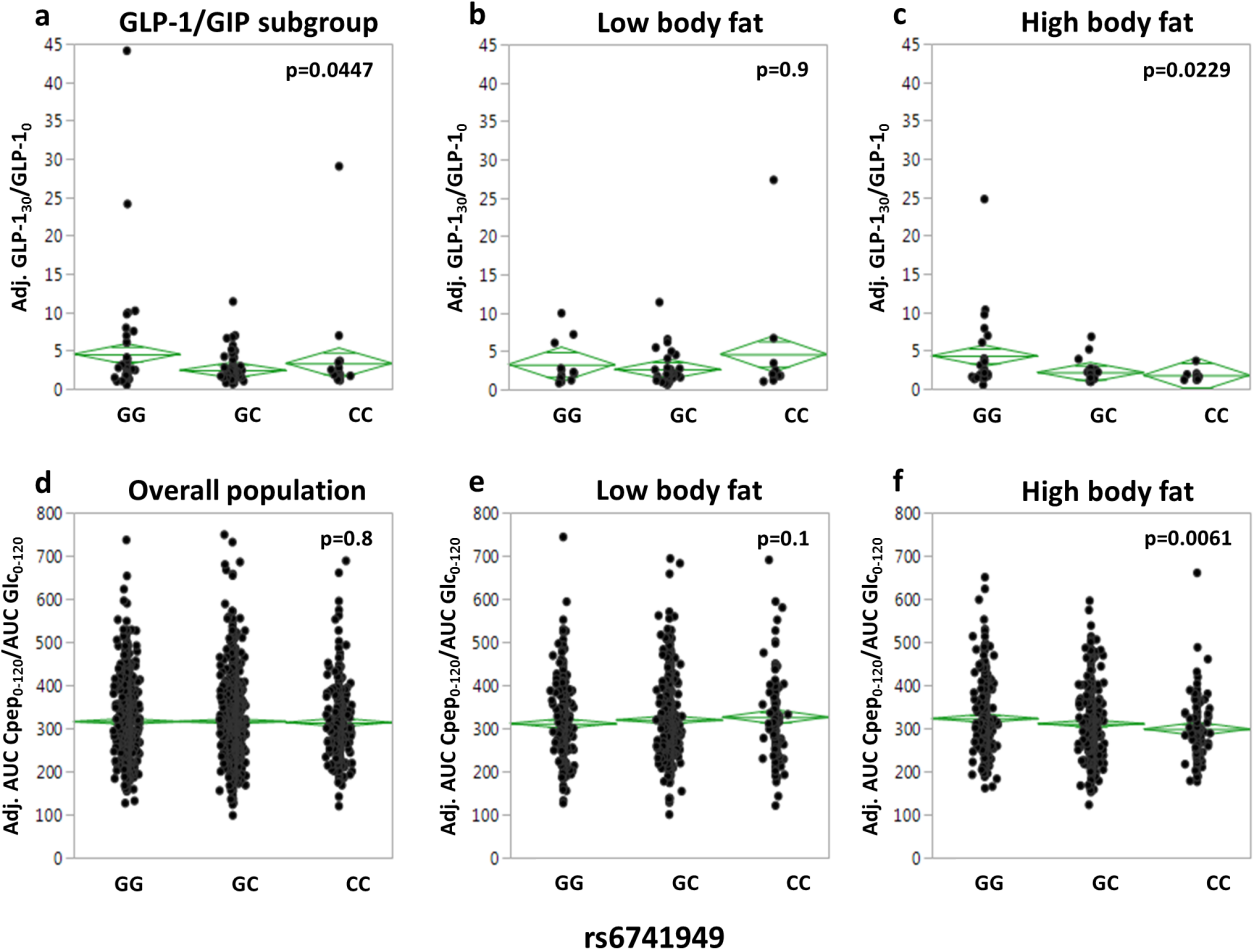
Association of *DPP4* SNP rs6741949 with GLP-1 levels and insulin secretion during an OGTT. GLP-1 levels (a-c) are presented as ratio of GLP-1 at 30 min of the OGTT divided by GLP-1 at baseline adjusted for gender, age, and, in the unstratified subgroup, bioimpedance-derived percentage of body fat. Insulin secretion (d-f) is presented as ratio of AUC C-peptide during the complete 2-h OGTT divided by AUC glucose (x10^-9^) adjusted for gender, age, OGTT-derived insulin sensitivity, and, in the unstratified overall population, percentage of body fat. For stratification, the population was divided into quintiles of percentage of body fat. The lowest two quintiles were combined to form the low body fat group (b and e), the highest two quintiles to form the high body fat group (c and f). The middle quintile was excluded. Individual data and mean diamonds (green) are plotted. The top and the bottom of the mean diamond mark the 95% confidence interval; the mean line across the middle of the diamond represents the group mean; and the lines above and below the group mean represent overlap marks. AUC–area under the curve; GLP-1 –glucagon-like peptide 1; OGTT–oral glucose tolerance test; SNP–single nucleotide polymorphism.

Initially, we screened all 14 tagging SNPs for association with fasting incretin levels and with incretin increments during the first 30 min of the OGTT as assessed by the ratios GLP-1_30_/GLP-1_0_ and GIP_30_/GIP_0_. After adjustment for gender, age, and percentage of body fat, no associations that resisted Bonferroni correction for multiple comparisons (p<0.0037) could be detected, and SNP rs6741949 was the only SNP to show a nominal association with oral glucose-stimulated GLP-1 increase (p = 0.0447; S3 Table and Fig 2A).

As DPP-4 was recently described to be an adipokine that is preferentially released by hypertrophic adipocytes [23], we then addressed whether any of the tagging SNPs demonstrates interaction effects with body fat on incretin levels. Using analysis of covariance with gender and age as covariates, only one SNP revealed significant interaction with bioimpedance-derived body fat content: it was SNP rs6741949 that showed an interaction effect on the oral glucose-stimulated GLP-1 increase (p = 0.0021; S3 Table). Notably, no interaction effect of SNP rs6471949 on the GLP-1 increment was seen when BMI was used instead of percentage of body fat (p = 0.8). None of the tagging SNPs showed association with fasting or glucose-stimulated GIP levels in these analyses (p≥0.07; S3 Table). Nominal interaction results of SNPs that were not associated with incretin levels in our initial analysis were not followed up.

To assess whether SNP rs6471949 reveals effects in subjects with high body fat content (i.e., with hypertrophic adipose tissue) only, we divided the overall study population and the GLP-1/GIP subgroup in body fat strata. Since genotype-trait interactions are often driven by the more extreme parts of a trait’s data distribution, we excluded the area around the median as “grey zone”. On the other hand, we intended to keep the excluded sample small in order not to lose statistical power. Therefore, we chose quintiles and stratified the subjects in a low body fat group (lowest two quintiles combined) and a high body fat group (highest two quintiles combined) and excluded the middle quintile from the analysis. In these follow-up analyses, the significance threshold was set at p<0.05. After adjustment for gender and age, the minor C-allele of SNP rs6741949 was associated with a lower glucose-induced GLP-1 increment in subjects with high, but not in subjects with low, body fat content (p = 0.0229 versus p = 0.9; Fig 2B and 2C). This result was unaffected by additional inclusion of glucose tolerance status in the multiple regression models (high body fat: p = 0.0284; low body fat: p = 0.9).

Next, we tested whether this SNP’s effect on GLP-1 is reflected by a concordant effect on insulin secretion in the overall study population. After adjustment for gender, age, body fat, and ISI OGTT, no SNP effect on insulin secretion was detectable in the unstratified study population (p = 0.8; Fig 2D). After stratification for body fat and adjustment for gender, age, and ISI OGTT, the minor C-allele of SNP rs6741949 showed the expected insulin secretion-impairing effect in the high, but not in the low, body fat group (p = 0.0061 versus p = 0.1; Fig 2E and 2F). Again, this result was unaffected by additional inclusion of glucose tolerance status in the multiple regression models (high body fat: p = 0.0275; low body fat: p = 0.2).

**Fig 3:**
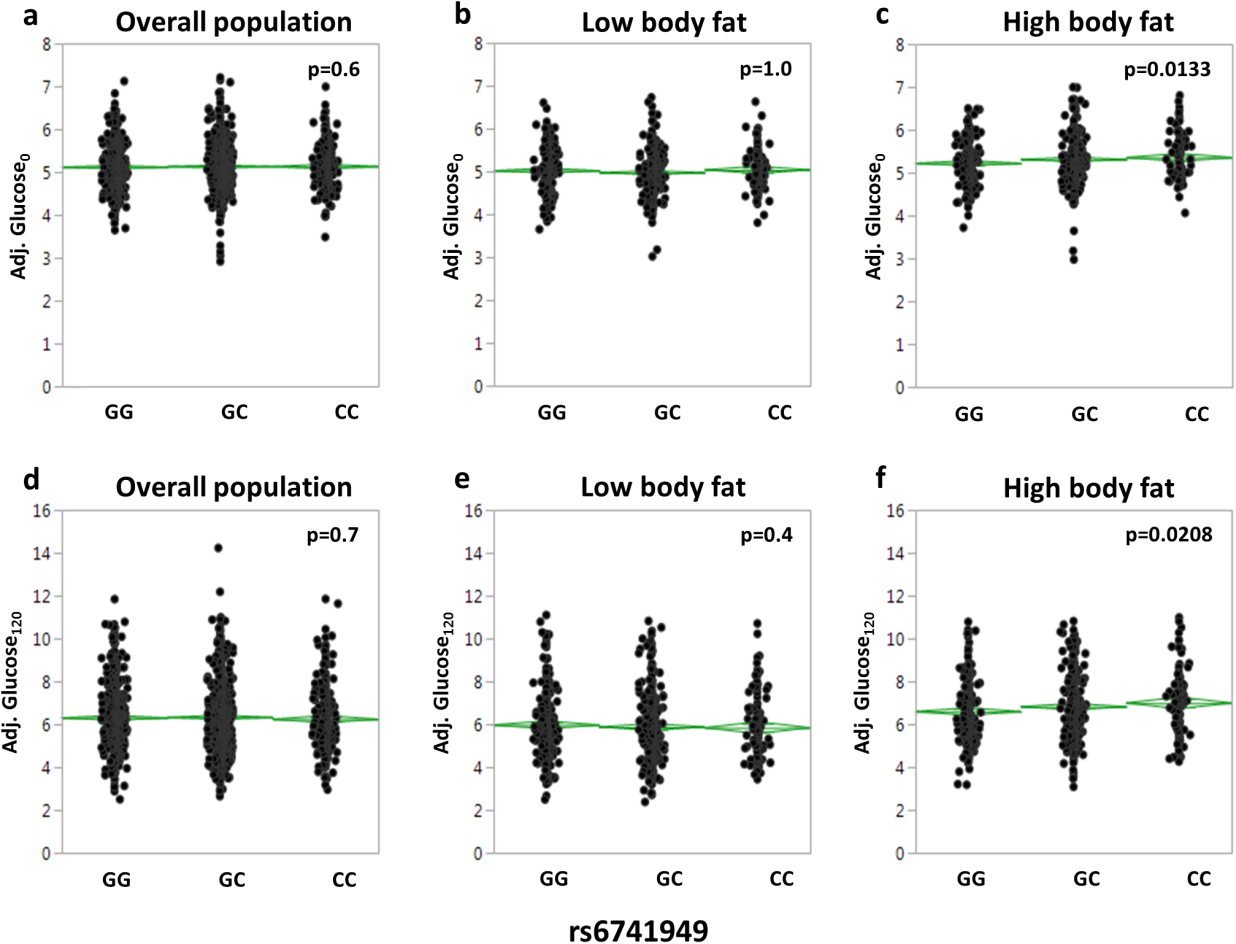
Association of *DPP4* SNP rs6741949 with fasting glucose levels and glucose levels at 120 min of the OGTT. Fasting glucose levels (in mmol/L; a-c) and 2-h glucose levels (in mmol/L; d-f) are presented after adjustment for gender, age, and, in the unstratified overall population, bioimpedance-derived percentage of body fat. For stratification, the population was divided into quintiles of percentage of body fat. The lowest two quintiles were combined to form the low body fat group (b and e), the highest two quintiles to form the high body fat group (c and f). The middle quintile was excluded. Individual data and mean diamonds (green) are plotted. The top and the bottom of the mean diamond mark the 95% confidence interval; the mean line across the middle of the diamond represents the group mean; and the lines above and below the group mean represent overlap marks. OGTT–oral glucose tolerance test; SNP–single nucleotide polymorphism.

Then, we asked whether the SNP additionally affects fasting glycaemia and/or glucose tolerance. Again, no effects on fasting or 2-h glucose levels were seen in the unstratified study population after adjustment for gender, age, and body fat (p = 0.6 and p = 0.7, respectively; Fig 3A and 3D). After stratification for body fat and adjustment for gender and age, the minor C-allele increased fasting and 2-h glucose levels in the high, but not in the low, body fat group (fasting glucose: p = 0.0133 versus p = 1.0; 2-h glucose: p = 0.0208 versus p = 0.6; Fig 3B, 3C, 3E and 3F).

Since SNP rs6741949 is intronic and may influence DPP-4 expression or mRNA stability, we measured fasting plasma DPP-4 levels in the GLP-1/GIP subgroup. However, we did not detect SNP-dependent differences in circulating DPP-4 levels either in the unstratified group (p = 0.5; adjusted for gender, age, and body fat) or in the low and high body fat strata (p≥0.1; adjusted for gender and age).

Finally, we interrogated publicly available data of the Meta-Analyses of Glucose and Insulin-related traits Consortium (MAGIC). In these unstratified datasets, SNP rs6741949 revealed weak trends for congruent association with three parameters of insulin secretion, i.e., insulin concentrations at 30 min of the OGTT (p = 0.06, N = 4,483), 30-min insulin concentrations adjusted for 30-min glucose concentrations and BMI (p = 0.09, N = 4,409), and incremental insulin concentrations 0–30 min (p = 0.08, N = 4,447). Data stratified for body adiposity were not available.

## Discussion

In this study, we identified by a tagging SNP approach a common intronic variant, i.e., SNP rs6741949, in the *DPP4* gene that negatively affects glucose-stimulated GLP-1 levels, insulin secretion, and glucose tolerance in humans. Most interestingly, the SNP effect was detectable in subjects with high body adiposity only, whereas no phenotypic differences were seen in subjects with low body adiposity. This is in agreement with the recent *in vitro* finding that DPP-4 represents an adipokine released by hypertrophic adipocytes [23], with the *in vivo* observation of higher DPP-4 levels/activity in obese rodent models [24] and humans [25–27], and with accordingly lower postprandial GLP-1 levels in obese subjects, as reported by two larger human studies [28,29]. Thus, only overweight and obese subjects may release sufficient DPP-4 to allow the SNP effect to become apparent. A lack of stratification for body fat content may also be the reason for the negative findings reported in an earlier *DPP4* tagging SNP study [30].

It is noteworthy that the SNP effects were only observed when we tested for genotype-body fat interaction using bioimpedance-derived percentage of body fat, but not when using BMI. The latter is probably an insufficient proxy for body fat content in this context. This limits the possibility to replicate our results since many pre-existing human studies of comparable sample size use BMI to assess body adiposity. Thus, novel studies with more precise body fat measurements are urgently needed. The use of BMI could also be the reason why a recent meta-analysis failed to detect a significant impact of body adiposity on the efficacy of vildagliptin [31].

As to the SNP’s role in different ethnicities, we can just speculate. The allele frequency of the minor C-allele considerably varies between the ethnicities analyzed in the 1000 Genomes Project (http://grch37.ensembl.org/Homo_sapiens/Info/Index), from 5% in Han Chinese to 50% in subjects from Los Angeles with Mexican ancestry. Moreover, the SNP exerts its effects predominantly in subjects with high body fat, and Chinese and Mexicans also considerably vary in the prevalence of obesity (~11% vs. ~30%, World Obesity Federation). Based on these data, we would expect that the SNP plays a more important role in Mexicans than in Han Chinese.

With respect to the functionality of the SNP, it has to be noted that the proglucagon gene (*GCG*) is adjacent and located ~70 kb upstream of the *DPP4* gene, and therefore, the SNP could theoretically influence the expression of proglucagon, the precursor of glucagon, GLP-1, and GLP-2. Thus, the SNP’s effect on GLP-1 formation/release could be a direct one. However, careful analysis of publicly available genetic linkage data (from the 1000 Genomes Project) revealed that no obvious linkage block spans both loci. Therefore, this possibility is rather unlikely, and the SNP might directly affect the *DPP4* gene.

A limitation of the study is that our blood samples were drawn and stored in the absence of DPP-4 inhibitors and thus were inappropriate to quantify the fraction of intact/active GLP-1 in addition to the total GLP-1 concentration. This measurement would have allowed the assessment of SNP rs6741949’s impact on DPP-4 activity. It is therefore also possible that the SNP affects GLP-1 secretion rather than inactivation by modulating DPP-4’s feedback effect on GLP-1-producing intestinal L-cells, a phenomenon recently discussed in [32]. Moreover, the SNP could have provoked a delay in GLP-1 secretion that was not detectable with our time-points of GLP-1 measurement (0, 30, and 120 min of the OGTT). Further investigations are required to assess these points.

Since the SNP is intronic, it might impact DPP-4 expression or mRNA stability rather than its enzymatic function. Therefore, we measured plasma DPP-4 concentrations in the subgroup that revealed sufficient statistical power to detect SNP effects on glucose-stimulated GLP-1 increase. However, we did not detect SNP-dependent differences in circulating DPP-4 levels. This could be due to the fact that DPP-4 is a ubiquitously expressed transmembrane protein and our measurement was restricted to the proteolytically generated circulating form of DPP-4. Clearly, the exact mechanism underlying the observed SNP effects requires further investigation. In particular, studies addressing the SNP’s effects on DPP-4 gene expression in sufficiently large collections of relevant tissues would help elucidate the SNP’s functionality. In addition, the SNP did not affect fasting or glucose-stimulated GIP levels. Since GLP-1 and GIP are cleaved by DPP-4 with comparable rates [3], this could reflect divergent feedback effects of DPP-4 on incretin secretion by L- versus K-cells. This aspect of GLP-1 selectivity needs further clarification as well.

Finally, we emphasize that this is not a pharmacogenetic study and, thus, cannot give an appropriate answer to the question whether SNP rs6741949 is causative for the reported biological variance in response to DPP-4 inhibitors. Based on the presented results, however, pharmacogenetic investigations addressing the effect of DPP-4 inhibitors on blood glucose and/or HbA1c levels as a function of the rs6741949 genotype would be promising and would have a good chance to increase our understanding of the biological causes of limited response/non-response to DPP-4 inhibitors.

In conclusion, we identified a common tagging SNP, i.e., rs6741949, in the *DPP4* gene that negatively affects glucose-stimulated GLP-1 secretion, insulin secretion, and glucose tolerance. This variant interacts with body fat, and its effects are unmasked by stratification for body adiposity. Even though the mechanism behind these effects remains to be established, common genetic variation in the *DPP4* gene tagged by SNP rs6741949 could possibly underlie the reported inter-individual variance in responsiveness to DPP-4 inhibitors, especially in subjects with high body fat content. If corroborated in respective pharmacogenetic studies, this gene variation could influence clinical decisions, e.g., regarding the use of DPP-4 inhibitors versus incretin mimetics, in terms of individualized therapy of type-2 diabetes.

## Supporting information

S1 Table Minor allele frequencies of the *DPP4* tagging SNPs observed in the overall study population compared to HapMap CEU data. (DOCX)

S2 Table Linkage disequilibrium data (D’, r^2^) of the *DPP4* tagging SNPs observed in the overall study population. (DOCX)

S3 Table Associations of the *DPP4* tagging SNPs with incretin levels (GLP-1/GIP subgroup). (DOCX)
