# Supplementary material for: DPP4 gene variation affects GLP-1 secretion, insulin secretion, and glucose tolerance in humans with high body adiposity
Source: PLoS One. 2017 Jul 27;12(7):e0181880. doi: 10.1371/journal.pone.0181880 (PMC5531535; doi:10.1371/journal.pone.0181880)
Supplement: S1 Table — (DOCX) [file pone.0181880.s001.docx]

**S1 Table. Minor allele frequencies of the *DPP4* tagging SNPs observed in the overall study population compared to HapMap CEU data**

| SNP | MAF overall cohort | MAF HapMap CEU |
| --- | --- | --- |
| rs2909443 | 0.394 | 0.416 |
| rs2909448 | 0.416 | 0.391 |
| rs2389643 | 0.111 | 0.121 |
| rs2909450 | 0.196 | 0.223 |
| rs1014444 | 0.375 | 0.367 |
| rs6432708 | 0.368 | 0.376 |
| rs12995983 | 0.264 | 0.257 |
| rs3788979 | 0.131 | 0.137 |
| rs6741949 | 0.405 | 0.434 |
| rs4664446 | 0.453 (G-allele) | 0.496 (A-allele) |
| rs741529 | 0.125 | 0.150 |
| rs3788976 | 0.259 | 0.257 |
| rs12469968 | 0.493 (G-allele) | 0.491 (A-allele) |
| rs1861978 | 0.303 | 0.317 |

CEU – Central Europeans; MAF – minor allele frequency; SNP – single nucleotide polymorphism
