# Supplementary material for: DPP4 gene variation affects GLP-1 secretion, insulin secretion, and glucose tolerance in humans with high body adiposity
Source: PLoS One. 2017 Jul 27;12(7):e0181880. doi: 10.1371/journal.pone.0181880 (PMC5531535; doi:10.1371/journal.pone.0181880)
Supplement: S2 Table — (DOCX) [file pone.0181880.s002.docx]

**S2 Table. Linkage disequilibrium data (D’, r²) of the *DPP4* tagging SNPs observed in the overall study population**

|  | rs2909443 | rs2909448 | rs2389643 | rs2909450 | rs1014444 | rs6432708 | rs12995983 | rs3788979 | rs6741949 | rs4664446 | rs741529 | rs3788976 | rs12469968 | rs1861978 |
| --- | --- | --- | --- | --- | --- | --- | --- | --- | --- | --- | --- | --- | --- | --- |
| rs2909443 | - | 0.965 | 0.979 | 0.931 | 0.840 | 0.966 | 0.961 | 0.953 | 0.757 | 0.775 | 0.548 | 0.587 | 0.205 | 0.377 |
| rs2909448 | 0.847 | - | 1.000 | 0.995 | 0.872 | 0.990 | 0.990 | 0.967 | 0.735 | 0.722 | 0.502 | 0.441 | 0.249 | 0.363 |
| rs2389643 | 0.184 | 0.175 | - | 1.000 | 1.000 | 0.975 | 0.974 | 1.000 | 0.938 | 0.919 | 0.946 | 0.796 | 0.781 | 0.782 |
| rs2909450 | 0.137 | 0.172 | 0.030 | - | 1.000 | 0.987 | 0.988 | 0.941 | 0.730 | 0.732 | 0.565 | 0.580 | 0.250 | 0.202 |
| rs1014444 | 0.274 | 0.324 | 0.075 | 0.146 | - | 0.982 | 0.988 | 0.980 | 0.806 | 0.615 | 0.531 | 0.421 | 0.450 | 0.481 |
| rs6432708 | 0.839 | 0.804 | 0.204 | 0.138 | 0.337 | - | 0.998 | 1.000 | 0.787 | 0.824 | 0.519 | 0.719 | 0.211 | 0.387 |
| rs12995983 | 0.511 | 0.494 | 0.330 | 0.085 | 0.210 | 0.613 | - | 1.000 | 0.780 | 0.777 | 0.374 | 0.672 | 0.030 | 0.192 |
| rs3788979 | 0.088 | 0.100 | 0.019 | 0.032 | 0.241 | 0.087 | 0.054 | - | 0.910 | 0.492 | 0.938 | 0.792 | 0.478 | 0.242 |
| rs6741949 | 0.546 | 0.510 | 0.162 | 0.088 | 0.264 | 0.530 | 0.321 | 0.084 | - | 0.807 | 0.531 | 0.541 | 0.262 | 0.425 |
| rs4664446 | 0.471 | 0.449 | 0.128 | 0.108 | 0.187 | 0.479 | 0.262 | 0.030 | 0.535 | - | 0.597 | 0.596 | 0.309 | 0.604 |
| rs741529 | 0.028 | 0.026 | 0.016 | 0.011 | 0.068 | 0.023 | 0.007 | 0.019 | 0.028 | 0.042 | - | 1.000 | 0.980 | 0.967 |
| rs3788976 | 0.078 | 0.049 | 0.028 | 0.235 | 0.037 | 0.105 | 0.057 | 0.033 | 0.070 | 0.103 | 0.050 | - | 0.409 | 0.963 |
| rs12469968 | 0.027 | 0.043 | 0.078 | 0.015 | 0.125 | 0.025 | <0.001 | 0.035 | 0.045 | 0.077 | 0.142 | 0.057 | - | 0.985 |
| rs1861978 | 0.095 | 0.080 | 0.033 | 0.004 | 0.060 | 0.111 | 0.030 | 0.004 | 0.115 | 0.192 | 0.058 | 0.141 | 0.410 | - |

D’-values are shown above empty cells, r²-values below empty cells. SNP – single nucleotide polymorphism
