# Supplementary material for: DPP4 gene variation affects GLP-1 secretion, insulin secretion, and glucose tolerance in humans with high body adiposity
Source: PLoS One. 2017 Jul 27;12(7):e0181880. doi: 10.1371/journal.pone.0181880 (PMC5531535; doi:10.1371/journal.pone.0181880)
Supplement: S3 Table — (DOCX) [file pone.0181880.s003.docx]

**S3 Table. Associations of the *DPP4* tagging SNPs with incretin levels (GLP-1/GIP subgroup)**

|  | Genotype | N | GLP-1_0_  OGTT (pmol/L) | GLP-1_30_/GLP-1_0_  OGTT (pmol/L) | GIP_0_  OGTT (pmol/L) | GIP_30_/GIP_0_  OGTT (pmol/L) |
| --- | --- | --- | --- | --- | --- | --- |
| rs2909443 | AA | 55 | 16.4 ±10.6 | 4.25 ±5.34 | 13.3 ±8.0 | 10.43 ±9.01 |
|  | AG | 89 | 17.9 ±7.8 | 2.29 ±1.37 | 14.2 ±8.0 | 8.25 ±6.75 |
|  | GG | 24 | 15.6 ±6.2 | 3.17 ±5.16 | 14.9 ±6.4 | 9.04 ±11.28 |
| p (p_interaction_) | - | - | 0.2 (0.06) | 0.05 (**0.0153**) | 0.5 (0.2) | 0.2 (0.1) |
|  | | | | | | |
| rs2909448 | TT | 50 | 16.7 ±10.7 | 4.30 ±5.55 | 13.1 ±8.3 | 10.92 ±9.33 |
|  | TC | 93 | 17.5 ±7.9 | 2.38 ±1.52 | 14.1 ±7.8 | 8.11 ±6.64 |
|  | CC | 25 | 16.2 ±6.4 | 3.10 ±5.06 | 15.3 ±6.5 | 9.05 ±11.01 |
| p (p_interaction_) | - | - | 0.2 (0.07) | 0.08 (**0.0187**) | 0.4 (0.1) | 0.2 (0.2) |
|  | | | | | | |
| rs2389643 | CC | 129 | 17.1 ±9.0 | 3.28 ±4.26 | 13.9 ±8.0 | 9.01 ±7.67 |
|  | CT | 35 | 17.1 ±7.4 | 2.27 ±1.41 | 14.5 ±6.8 | 9.41 ±10.66 |
|  | TT | 4 | 16.8 ±5.3 | 2.20 ±1.09 | 14.3 ±9.8 | 7.97 ±5.85 |
| p (p_interaction_) | - | **-** | 0.4 (1.0) | 0.2 (0.9) | 0.7 (0.5) | 0.8 (0.7) |
|  | | | | | | |
| rs2909450 | GG | 103 | 17.7 ±8.7 | 2.80 ±3.99 | 14.6 ±8.0 | 8.62 ±7.92 |
|  | GA | 60 | 15.2 ±7.9 | 3.59 ±3.61 | 12.8 ±7.2 | 10.12 ±9.18 |
|  | AA | 5 | 26.0 ±8.3 | 2.01 ±1.12 | 16.8 ±7.3 | 6.30 ±2.99 |
| p (p_interaction_) | - | **-** | 0.5 (0.4) | 0.4 (0.5) | 0.8 (0.3) | 0.9 (0.8) |
|  | | | | | | |
| rs1014444 | AA | 68 | 17.3 ±6.6 | 2.57 ±3.26 | 14.5 ±6.9 | 8.54 ±8.63 |
|  | AG | 82 | 16.4 ±9.3 | 3.28 ±3.27 | 13.6 ±8.2 | 9.72 ±8.44 |
|  | GG | 18 | 19.4 ±11.4 | 3.74 ±6.92 | 14.1 ±9.1 | 8.05 ±6.25 |
| p (p_interaction_) | - | - | 0.3 (0.1) | 0.1 (0.09) | 0.3 (0.2) | 0.5 (0.3) |

(continued)

|  | Genotype | N | GLP-1_0_  OGTT (pmol/L) | GLP-1_30_/GLP-1_0_  OGTT (pmol/L) | GIP_0_  OGTT (pmol/L) | GIP_30_/GIP_0_  OGTT (pmol/L) |
| --- | --- | --- | --- | --- | --- | --- |
| rs6432708 | TT | 60 | 16.6 ±10.6 | 4.12 ±5.14 | 13.1 ±8.0 | 9.98 ±8.82 |
|  | TC | 87 | 17.8 ±7.5 | 2.25 ±1.33 | 14.4 ±7.9 | 8.34 ±6.74 |
|  | CC | 21 | 15.6 ±6.5 | 3.35 ±5.51 | 14.9 ±6.6 | 9.53 ±11.98 |
| p | - | - | 0.1 (**0.0642**) | 0.06 (**0.0091**) | 0.3 (0.07) | 0.5 (0.4) |
|  | | | | | | |
| rs12995983 | TT | 90 | 17.1 ±9.8 | 3.39 ±4.32 | 13.8 ±8.3 | 9.50 ±8.39 |
|  | TC | 65 | 17.4 ±7.1 | 2.69 ±3.39 | 14.3 ±7.0 | 7.93 ±5.99 |
|  | CC | 13 | 14.8 ±5.8 | 2.49 ±1.08 | 14.3 ±8.2 | 11.78 ±14.97 |
| p (p_interaction_) | - | - | 0.3 (0.1) | 0.5 (**0.0241**) | 0.8 (0.2) | 0.9 (0.4) |
|  | | | | | | |
| rs3788979 | GG | 128 | 16.7 ±7.7 | 2.97 ±3.45 | 13.8 ±7.5 | 8.93 ±8.29 |
|  | GA | 38 | 17.8 ±8.7 | 2.59 ±1.78 | 14.2 ±8.5 | 9.80 ±8.49 |
|  | AA | 2 | 29.0 ±39.6 | 16.11 ±21.06 | 22.0 ±11.3 | 3.50 ±2.32 |
| p | - | **-** | 1.0 (1.0) | 0.2 (0.1) | 0.2 (0.7) | 0.7 (0.2) |
|  | | | | | | |
| rs6741949 | GG | 50 | 16.3 ±10.4 | 4.22 ±5.39 | 13.6 ±8.3 | 10.11 ±8.57 |
|  | GC | 94 | 17.7 ±7.8 | 2.40 ±1.67 | 13.7 ±7.0 | 8.56 ±7.24 |
|  | CC | 24 | 16.4 ±7.3 | 3.14 ±5.25 | 16.0 ±9.3 | 8.88 ±11.23 |
| p (p_interaction_) | - | **-** | 0.2 (**0.0131**) | **0.0447** (**0.0021**) | 0.4 (0.5) | 0.1 (0.3) |
|  | | | | | | |
| rs4664446 | AA | 49 | 15.5 ±8.7 | 4.24 ±5.62 | 13.0 ±8.2 | 10.10 ±8.01 |
|  | AG | 87 | 18.5 ±9.2 | 2.40 ±1.52 | 13.9 ±7.3 | 8.69 ±7.90 |
|  | GG | 32 | 15.5 ±6.0 | 3.02 ±4.54 | 15.8 ±8.2 | 8.55 ±9.74 |
| p (p_interaction_) | - | - | 0.2 (0.2) | 0.2 (0.07) | 0.1 (0.2) | 0.2 (0.4) |
|  | | | | | | |
| rs741529 | GG | 134 | 17.8 ±8.9 | 3.11 ±4.09 | 13.5 ±7.6 | 9.29 ±8.81 |
|  | GA | 27 | 14.7 ±6.7 | 2.69 ±2.47 | 16.7 ±8.3 | 7.53 ±5.43 |
|  | AA | 4 | 10.8 ±6.8 | 3.34 ±2.67 | 7.8 ±6.0 | 12.49 ±7.25 |
| p (p_interaction_) | - | - | 0.1(0.4) | 1.0 (0.3) | 1.0 (0.7) | 0.5 (0.8) |

(continued)

|  | Genotype | N | GLP-1_0_  OGTT (pmol/L) | GLP-1_30_/GLP-1_0_  OGTT (pmol/L) | GIP_0_  OGTT (pmol/L) | GIP_30_/GIP_0_  OGTT (pmol/L) |
| --- | --- | --- | --- | --- | --- | --- |
| rs3788976 | CC | 92 | 17.2 ±8.9 | 2.87 ±3.38 | 14.3 ±8.0 | 9.08 ±8.56 |
|  | CT | 64 | 16.7 ±7.8 | 3.32 ±4.57 | 13.7 ±7.7 | 9.28 ±8.45 |
|  | TT | 12 | 17.6 ±11.0 | 2.92 ±2.51 | 13.1 ±6.5 | 7.83 ±5.25 |
| p (p_interaction_) | - | - | 0.6 (1.0) | 0.7 (1.0) | 0.5 (0.7) | 0.7 (0.3) |
|  | | | | | | |
| rs12469968 | AA | 50 | 16.8 ±9.2 | 3.70 ±4.83 | 13.0 ±6.6 | 8.47 ±7.16 |
|  | AG | 82 | 17.8 ±8.7 | 2.73 ±3.62 | 14.7 ±8.4 | 9.80 ±9.50 |
|  | GG | 36 | 15.7 ±7.3 | 2.84 ±2.26 | 14.0 ±7.8 | 8.18 ±6.62 |
| p (p_interaction_) | - | **-** | 0.7 (0.2) | 0.5 (0.05) | 0.6 (0.8) | 0.9 (0.8) |
|  | | | | | | |
| rs1861978 | TT | 76 | 16.5 ±8.3 | 3.05 ±3.95 | 14.2 ±8.0 | 8.56 ±6.66 |
|  | TG | 76 | 18.3 ±8.9 | 2.63 ±2.73 | 13.9 ±8.1 | 9.90 ±10.26 |
|  | GG | 16 | 13.5 ±7.4 | 5.00 ±6.51 | 13.9 ±4.3 | 7.40 ±2.23 |
| p (p_interaction_) | - | **-** | 0.8 (0.2) | 0.3 (**0.0254**) | 0.5 (0.9) | 1.0 (0.8) |

Data are shown as unadjusted raw data (means ±SD). Prior to statistical analysis, incretin levels were adjusted for gender, age, and bioimpedance-derived percentage of body fat. For analysis of SNP-body fat interaction effects on incretin levels, cross effects were tested using multiple linear regression models with gender and age as covariates. p_interaction_ – p-value for SNP-body fat interaction. Nominal associations are marked by bold fonts, significant associations by bold fonts and underlining. GIP – gastric inhibitory polypeptide; GLP-1 – glucagon-like peptide 1; OGTT – oral glucose tolerance test; SNP – single nucleotide polymorphism
